# Novel Homozygous *PADI6* Variants in Infertile Females with Early Embryonic Arrest

**DOI:** 10.3389/fcell.2022.819667

**Published:** 2022-04-01

**Authors:** Yao Xu, Rongxiang Wang, Zhi Pang, Zhiyun Wei, Lihua Sun, Sa Li, Guanghua Wang, Yu Liu, Yiwen Zhou, Hongjuan Ye, Liping Jin, Songguo Xue

**Affiliations:** ^1^ Shanghai Key Laboratory of Maternal Fetal Medicine, Clinical and Translational Research Center, Shanghai First Maternity and Infant Hospital, School of Medicine, Tongji University, Shanghai, China; ^2^ Reproductive Medicine Center, Shanghai East Hospital, School of Medicine, Tongji University, Shanghai, China; ^3^ Liver Cancer Institute, Zhongshan Hospital, Key Laboratory of Carcinogenesis and Cancer Invasion, Ministry of Education, Fudan University, Shanghai, China; ^4^ Key Laboratory of Receptor Research, Shanghai Institute of Materia Medica, University of Chinese Academy of Sciences, Beijing, China

**Keywords:** female infertility, early embryonic arrest, PADI6, novel variant, subcortical maternal complex

## Abstract

Early embryonic arrest denotes premature termination of development in preimplantation embryos, which is one of the major phenotypes of recurrent assisted reproduction failure. Padi6 is proven to be a member of the subcortical maternal complex (SCMC) in mice, which is essential in oocyte maturation and embryogenesis. We and other groups previously found that biallelic mutations in *PADI6* caused female infertility manifesting as early embryonic arrest. In this study, we identified two novel homozygous variants (p.Cys163Arg, and p. Trp475*) of *PADI6* in two infertile patients from a cohort of 75 females with the phenotype of early embryonic arrest. An *in vitro* expression study indicated severe decrease of PADI6, which might destruct the stability of SCMC. Our study expands the mutational spectrum of *PADI6* and further supports the causality between *PADI6* mutations and female infertility.

## Introduction

The prevalence of infertility is estimated to be 8%–12% among reproductive-aged couples worldwide (Vander Borght and Wyns, 2018). The increasing number of infertile people accelerates the process of an aging population, which has become a serious social issue (Datta et al., 2016). With the help of assisted reproductive technology (ART), millions of couples with fertility problems deliver babies after 1–2 cycles of treatment. However, there is still a large number of people suffering from unexplained recurrent ART failure, among which early embryonic arrest accounts for a considerable proportion.

Early embryonic arrest is characterized by premature termination of development in preimplantation embryos. Its genetic basis was largely unknown for a long time. In the recent 5 years, with the advance of high-throughput sequencing technology, a group of pathogenic genes were identified, and biallelic mutations in *PADI6* (610363), *NLRP2* (609364), *NLRP5* (609658), and *FBXO43* (609110) were gradually reported to be responsible for the phenotype (Xu et al., 2016; Mu et al., 2019; Wang et al., 2021). *PADI6*, *NLRP2*, and *NLRP5* are considered to form a subcortical maternal complex (SCMC), a multiprotein complex first identified in mice, which plays a critical role in essential events during the oocyte-to-embryo transition, including maternal RNA metabolism, mitochondrial rearrangement, and zygote genome activation (ZGA) (Lu et al., 2017). In 2016, we first reported biallelic mutations in *PADI6* causing a lack of PADI6 in oocytes, which impaired the ZGA process of early embryonic development (Xu et al., 2016). Subsequently, studies from three independent groups strengthened the correlation between *PADI6* variants and early embryonic arrest (Wang et al., 2018; Zheng et al., 2020; Liu et al., 2021). However, despite the previous findings, the pathogeny of the majority of patients was still unclear, and the genetic basis of human early embryonic development remained to be investigated.

Here, we recruited 75 Chinese patients diagnosed with early embryonic arrest in addition to the subjects studied in 2016 and identified two novel homozygous variants in *PADI6* (p.Cys163Arg, and p. Trp475*). We investigated and compared the effects of the two variants with our previously identified variants (p.His211Gln, p. Gln324*, p. Gln381*, and p. Glu670Glyfs*48). Our study broadens the mutational spectrum of *PADI6* and provides the further relationship between *PADI6* variants and female infertility.

## Materials and Methods

### Patients

A total of 75 Han Chinese patients undergoing *in vitro* fertilization (IVF) or intracytoplasmic sperm injection (ICSI) treatment with the phenotype of recurrent early embryonic arrest were recruited from the Reproductive Medicine Center of Shanghai East Hospital and Shanghai First Maternity and Infant Hospital affiliated with the Tongji University School of Medicine. The cohort involved one consanguineous family and 74 nonconsanguineous families. All the patients were under 40 years of age, to whom written informed consent were provided. This study was approved by the ethical committee of Shanghai East Hospital and Shanghai First Maternity and Infant Hospital affiliated with Tongji University School of Medicine.

### Genetic Analysis

Genomic DNA of all participants was extracted from peripheral blood using the HiPure Blood DNA Mini Kit (Magen Biotechnology, China) and was sheared to acquire 150–200 bp fragments by Biorupter (Diagenode, Belgium). The ends repair and adaptor ligation was performed using the Fast Library Prep Kit (iGeneTech, China). Whole exons were captured with the AIExome Enrichment Kit V1 (iGeneTech, China), and sequencing was performed on the Illumina platform. FastQC filtered clean reads were mapped to the reference genome (hg19) using Bwa. After removing duplications, SNV and InDel were called and annotated using GATK. For the patient from the consanguineous family, homozygosity mapping was performed to identify homozygous regions using HomozygosityMapper (Seelow et al., 2009). Variants were filtered according to the following criteria: 1) not recorded or recorded to have a frequency less than 0.1% in four public databases (000 Genomes Project [1,000 g], ExAC, gnomAD, and the NHLBI Exome Sequencing Project Exome Variant Server [ESP6500]); 2) missense, nonsense, splice-site, and indel variants; 3) homozygous variants in the identified homozygous regions; 4) predicted to be damaging by at least two *in silico* prediction models (SIFT, PROVEAN, PolyPhen-2); 5) genes were highly or specifically expressed in oocytes and early embryos based on our in-house database; 6) genes previously reported to affect female fertility in human or mouse. Candidate genes were validated by Sanger sequencing. The co-segregation validated genes were then screened in the sporadic cases in our cohort.

### Conservation Analysis and Molecular Modeling

Multiple sequence alignment of the nearby amino acid sequence of Cys163 and Trp475 was performed using ClustalW (https://www.genome.jp/tools-bin/clustalw). The result was displayed using ESPript 3.0 (https://espript.ibcp.fr/ESPript/cgi-bin/ESPript.cgi) (Robert and Gouet, 2014). The wild-type model of PADI6 3D structure was generated based on the predicted result of AlphaFold Protein Structure database (https://www.alphafold.ebi.ac.uk/entry/Q6TGC4) (Jumper et al., 2021). The variant was mapped onto the atomic model using PyMol software.

### Plasmid Construction and Mutagenesis

The wild-type *PADI6* coding sequence was cloned and inserted into the pRK7 vector with the N-terminal Flag tag. The mutant plasmids were generated using the Mut Express MultiS Fast Mutagenesis Kit (Vazyme Biotech Co.,Ltd, China). All the constructs were confirmed by Sanger sequencing.

### Cell Culture and Transfection

Human embryonic kidney 293T (HEK-293T) cells were cultured in Dulbecco’s Modified Eagle’s Medium (Sigma-Aldrich) with 10% fetal bovine serum and 1% penicillin/streptomycin (Gibco). Transient transfections were performed using the PolyJet DNA Transfection Reagent (SignaGen Laboratories, United States) according to the manufacturers’ instructions. The pEGFP-C1 plasmid was co-transfected as controls. Three independent transfections were performed as the biological replicates.

### RNA Isolation and Quantitative RT-PCR

Total RNA was isolated from 293T cells 36 h after transfection using TRIzol reagent, and 500 ng RNA was used for reverse transcription using the Evo M-MLV RT Mix Kit (Accurate Biotechnology, AG11728, China). Quantitative RT-PCR was performed using the SYBR Green Premix Pro Taq HS qPCR Kit (Accurate Biotechnology, AG11718, China) and QuantStudio 5 Real-Time PCR Systems (Thermo Fisher Scientific). All reactions were performed in at least biological triplicates with technical duplicates. The primers used in the experiments are listed in Supplementary Table S1. The relative expression level was calculated by 2^−ΔΔCt^ normalized by endogenous GAPDH expression.

### Western Blot Analysis

Forty-eight hours after transfection, cells were harvested and lysed with RIPA lysis buffer containing 1% protease inhibitor cocktail (Bimake, United States). After 30 min incubating at 4°C, protein lysates were denatured in SDS protein loading buffer. Protein samples were separated in 10% sodium dodecyl sulfate-polyacrylamide gel electrophoresis and transferred onto nitrocellulose membranes (Millipore, United States). The membranes were blocked in 5% skim milk (FUJIFILM Wako Pure Chemical Corporation, Japan) and incubated at 4°C overnight with primary antibodies against Flag (1:3,000, 14793, Cell Signaling Technology) or Vinculin (1:5,000, ab129002, Abcam). After 2 h incubation with secondary antibodies (1:5,000, Abmart), The membranes were visualized using the FluorChem E system (ProteinSimple, United States).

## Results

### Identification of Novel Variants in *PADI6*

**FIGURE 1:**
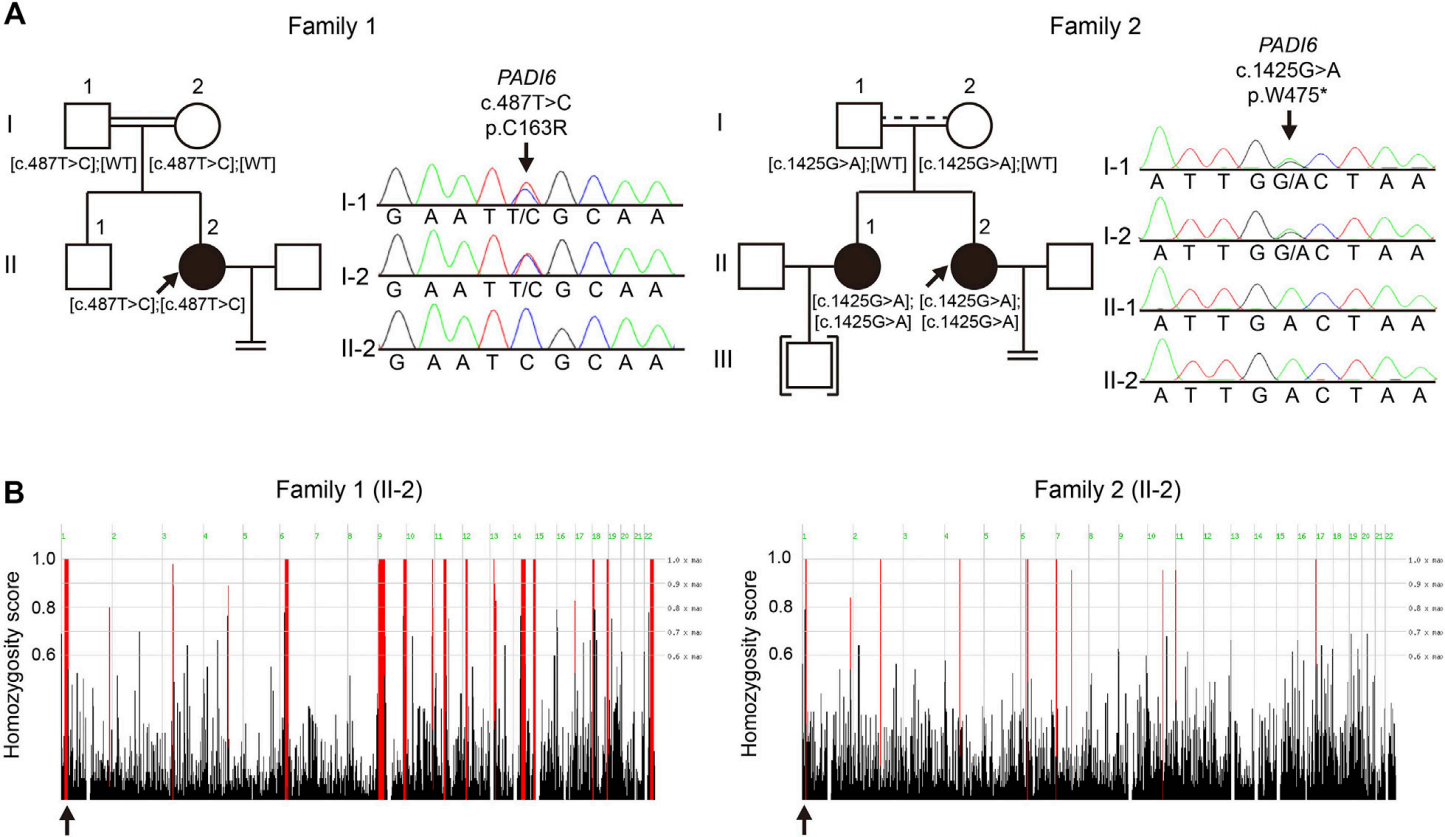
Identification of novel *PADI6* variants in two Chinese families with early embryonic arrest. **(A)** Pedigrees and Sanger sequencing results of the families. Black solid circles denote the patients; black arrows denote probands of the families. Brackets denote adoption, equal signs indicate infertility. Parallel double solid lines represent consanguinity; parallel solid line with dash line represent suspected consanguinity. **(B)** Homozygosity mapping of the probands in two families. Homozygous regions are indicated in red, and the black arrows indicate the location of *PADI6*.

**FIGURE 2:**
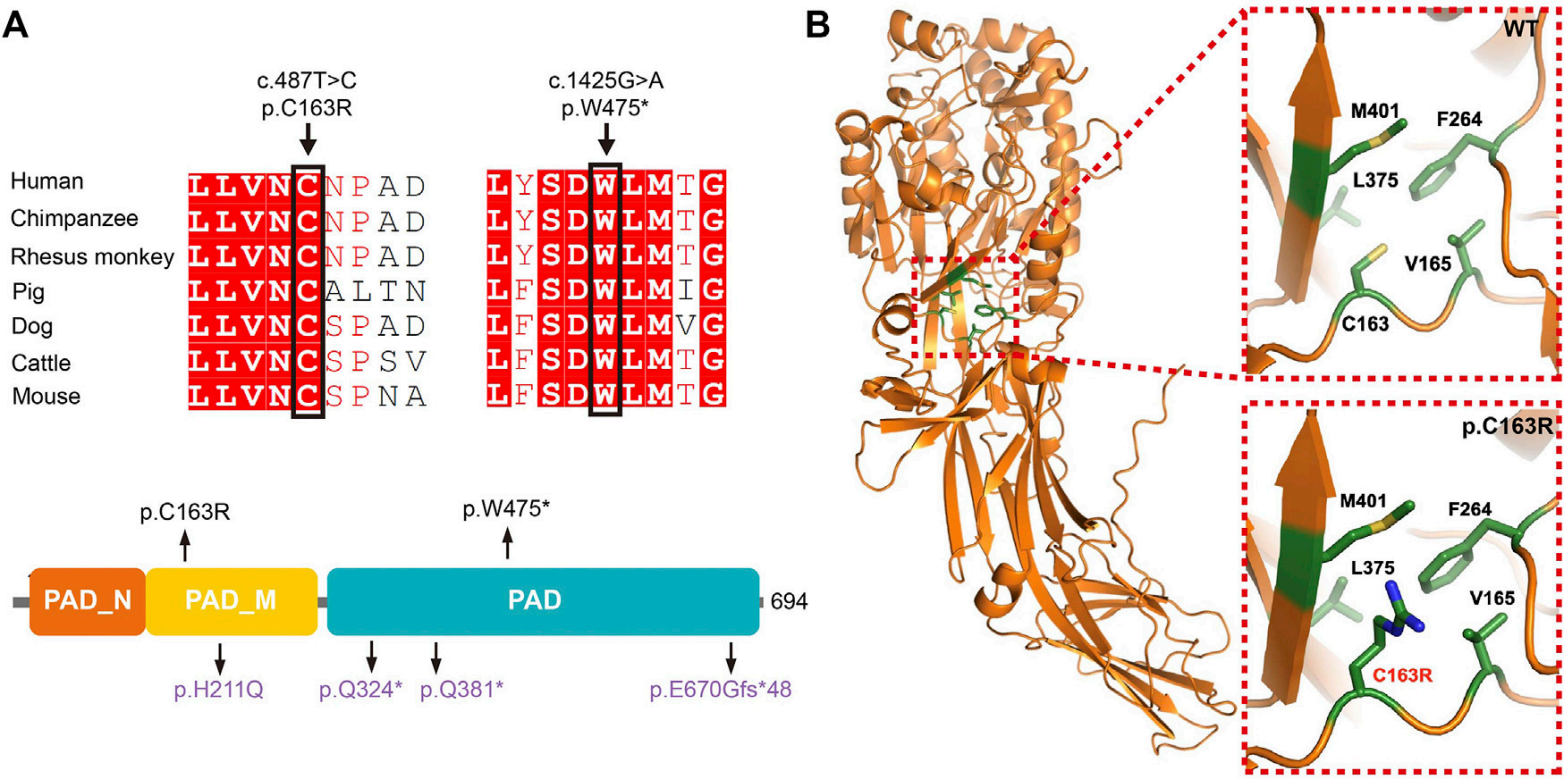
Location, conservation, and structure analysis of the mutant residues of PADI6. **(A)** Conservation analysis, and location of the mutant residues identified in our studies. Variants identified in this study are presented in black, and variants identified in our previous study are presented in purple. **(B)** Structure prediction of the p. Cys163Arg variant.

Whole-exome sequencing (WES), combined with homozygosity mapping, was first performed on the patient from the consanguineous family. Variant filtering was then conducted following our criteria. The first three criteria excluded most of the variants, and only 11 variants were retained in the gene list. After the *in silico* prediction procedure, four homozygous variants remained: c. 487T > C [p.Cys163Arg] in *PADI6* (NM_207421.4), c.524C > T [p.Pro175Leu] in *EPHA8* (MIM: 176945, NM_020526.5), c.688A > C [p.Lys230Gln] in *HAUS6* (MIM: 613433, NM_017645.5), and c.1559A > C [p.His520Pro] in *SLCO1B3* (MIM: 605495, NM_019844.4). *PADI6* is the only gene with high expression in oocytes and early stage embryos and was previously reported to associate with reproduction (Supplementary Figures S1A,B) (Yan et al., 2013). Sanger sequencing was subsequently performed to confirm the transmission pattern of the variant: The patient was homozygous, and her parents were heterozygous for the *PADI6* variant (Figure 1A, left). The variant was located in a 3.2 Mb homozygous region (Chr1:17235196-20510690, Figure 1B, left), and was absent in ExAC, gnomAD, ESP6500, and 1000G database (Table 1). It was predicted to be damaging or deleterious by SIFT, PROVEAN, and PolyPhen-2 (Table 1). The mutant amino acid was located in the PAD middle domain of PADI6 and was highly conserved among different species (Figure 2A).

**TABLE 1:** Overview of the *PADI6* variants identified from the patients.

Position	Sequence variation	Amino acid variation	Variant type	1000G_eas	ExAC_eas	gnomeAD	SIFT	PROVEAN	PolyPhen-2
chr1: 17707593	c.T487C	p.C163R	Missense	NA	NA	NA	Damaging	Deleterious	Probably damaging
chr1: 17721534	c.G1425A	p.W475*	Stop gain	NA	NA	NA	NA	Deleterious	NA

1000G_eas: allele frequency of variants in the East Asian population of the 1,000 Genomes Project; ExAC_eas: allele frequency of variants in the East Asian population of the Exome Aggregation Consortium (ExAC) browser; gnomAD: allele frequency of variants in the Genome Aggregation database; NA: not available.

We subsequently screened exons of *PADI6* in the cohort of 74 sporadic patients and identified one homozygous c.1425G > A [p.Trp475*] variant in *PADI6* (Figure 1A, right). Surprisingly, the patient denied that she was from a consanguineous family when we provided genetic counseling before she was included in our study. To confirm the causality and clarify the inheritance pattern of the variant, we first performed Sanger sequencing and confirmed the heterozygosity of her parents (Figure 1A, right). Then, we performed WES and homozygosity mapping on the patient, and the result indicated that her parents might have a consanguineous marriage, and the variant was located in a 2 Mb homozygous region on chromosome 1 (Chr1:17517064-19452948, Figure 1B, right). None of the previously reported causative gene variants were identified in the WES data. The variant was absent in the four public databases and was predicted to be deleterious by PROVEAN (Table 1). The Trp475 and the nearby residues were highly conserved among species (Figure 2A).

### *In Silico* Prediction of the Variant Effects on Protein Function

The p. Trp475* variant was located in the functional protein-arginine deiminase (PAD) domain, and the p. Cys163Arg variant was located in the PAD middle domain of PADI6 (Figure 2A). These two variants were located in the highly conserved regions among different species (Figure 2A). The p. Cys163Arg variant was predicted to be damaging or deleterious by SIFT, PROVEAN, and PolyPhen-2, and the p. Trp475* variant was predicted to be deleterious by PROVEAN (Table 1). According to the AlphaFold predicted PADI6 structure, the wild-type Cys163 was located in the core region of the protein. Arginine substitution produced a prolonged side chain with a positive charge, which might disrupt the surrounding environment and result in a conformational change and misfolding of protein (Figure 2B).

### Clinical Phenotypes of the Patients

**FIGURE 3:**
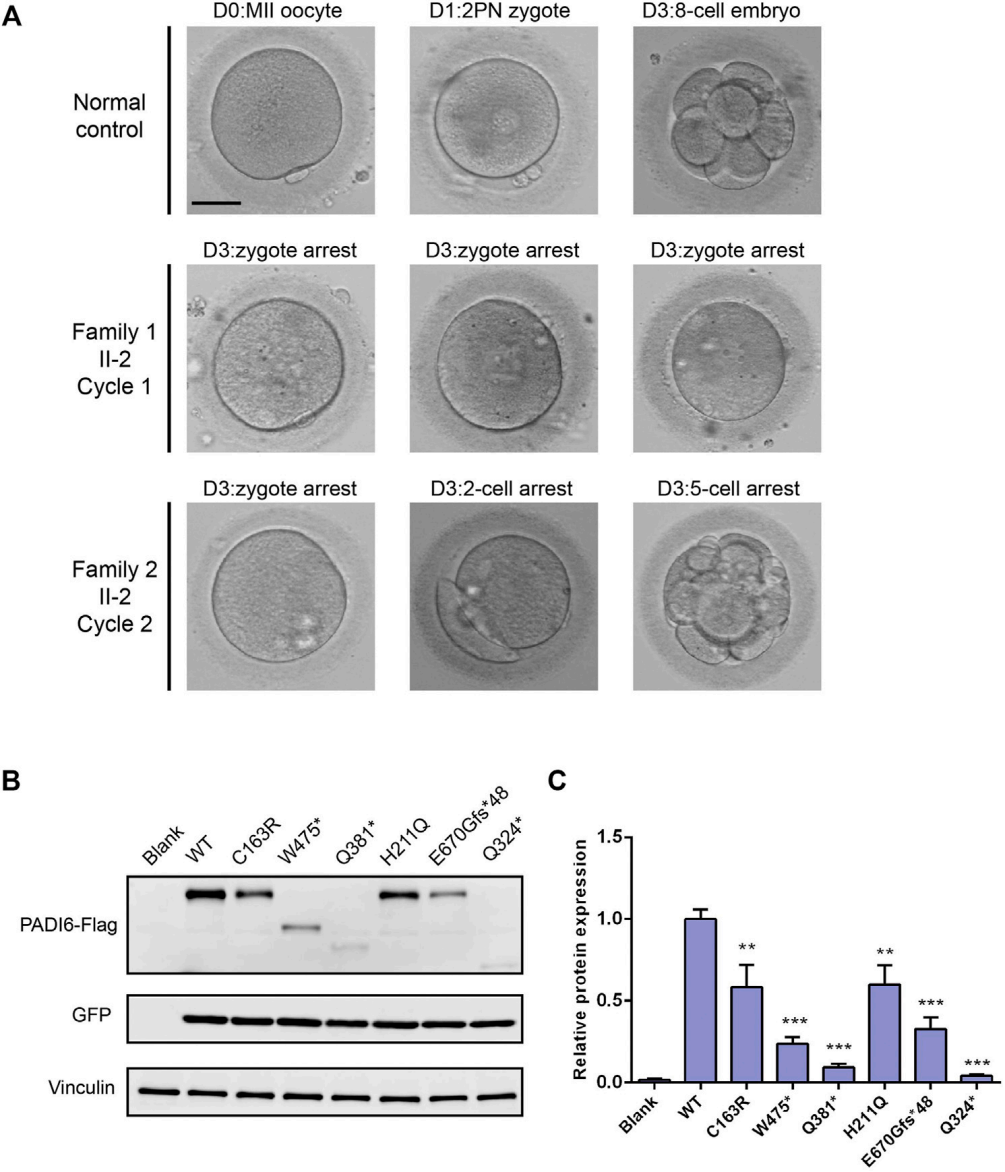
Effects of the variants on embryo development and protein expression. **(A)** Clinical phenotype of embryos from patients and control. Scale bar = 40 µm. **(B)** Western blot analysis of PADI6 protein expression in HEK-293T cells transfected with wild-type and mutant plasmids. **(C)** Quantitative analysis of wild-type and mutant PADI6 protein level. N = 3 independent transfections. ^**^, *p* <.01; ^***^, *p* <.001.

The patient from family 1 was from a consanguineous family. She was 33 years old and was diagnosed with primary infertility 6 years ago. She had experienced two failed ART attempts. In the first ART cycle, six oocytes were retrieved, and four matured oocytes were injected with sperm. Three oocytes were fertilized normally but were arrested at the zygote stage (Figure 3A). In the second cycle, one MII oocyte was retrieved and was inseminated by ICSI. However, only one pro-nuclear (PN) was observed on day 1, and the egg was discarded (Table 2).

**TABLE 2:** Clinical characteristic of the patients.

Patient	Age	Infertility years	ART cycles	Total oocytes	MII oocytes	2 PN zygotes	Abnormal fertilization zygotes	Normal cleavage embryos	Usable embryos on day 3
Family 1	33	6	1st ICSI	6	4	3	1	0	0
			2nd ICSI	1	1	0	1	0	0
Family 2	29	5	1st ICSI	15	13	7	6	7	2
			2nd ICSI	29	26	19	7	16	0

MII: metaphase II; 2 PN: two pronuclear.

The patient from family 2 was 29 years old and had not conceived with regular unprotected sexual intercourse for 5 years. During the ART treatment, a total of 44 oocytes were retrieved, and 39 MII oocytes were inseminated by ICSI. Among them, 26 oocytes were fertilized and cleaved normally. However, only two usable embryos were acquired on day 3, and most of the embryos were arrested before the eight-cell stage (Figure 3A). It is noteworthy that the patient has a sister diagnosed with primary infertility for several years, and the infertile couple eventually adopted a child after the failure of several years of pregnancy attempt. We performed Sanger sequencing on the sister and identified the homozygous c.1425G > A variant in *PADI6* (Figure 1A, right, II-1).

### *PADI6* Variants Affected Protein Expression in HEK-293T Cells

We constructed *PADI6* wild-type and mutant plasmids (two variants identified in this study and four variants identified in our previous study), and transiently transfected the constructs into HEK-293T cells to evaluate the functional influences of *PADI6* variants *in vitro*. Immunoblot examination showed that, compared with wild-type PADI6, the expression level of p. Cys163Arg protein was significantly reduced. The p. Trp475* protein was observed at the expected smaller size with significantly reduced expression (Figures 3B,C). The variants identified in our previous study displayed the same reduction in protein expression level, which was consistent with our previous findings in patients’ oocytes (Figures 3B,C) (Xu et al., 2016). Quantitative RT-PCR showed a significant reduction in mRNA levels in p. Trp475*, p. Gln324*, and p. Gln381* variants (Supplementary Figure S2).

## Discussion

In this study, we identified two novel homozygous variants (c.487T > C [p.Cys163Arg], and c.1425G > A [p.Trp475*]) in *PADI6* in two infertile patients from a cohort of 75 females with the phenotype of early embryonic arrest, which caused recurrent ART failure. We transfected mutant constructs in HEK-293T cells to investigate the potential functional influences caused by the two variants. The result suggests that the two variants could cause significant reduction in protein expression *in vitro*, which is similar to the pathogenic variants identified in our previous study.

PADI6 is a member of the peptidylarginine deiminase (PADI) family, a family of calcium-dependent post-translational modify enzymes that can catalyze the process of citrullination by converting positively charged arginine residues to neutrally charged citrulline (Horibata et al., 2012). The PADI family includes five isozymes, PADI1, PADI2, PADI3, PADI4, and PADI6. Each PADI has biased expression among different tissues and has been proven to play critical roles in multiple diseases, including rheumatoid arthritis, Alzheimer’s disease, ulcerative colitis, and cancers (Horibata et al., 2012; Mondal and Thompson, 2019; Curran et al., 2020; Al-U’datt et al., 2021). PADI6 is uniquely and highly expressed in different stages of oocytes and cleavage stage embryos, which suggests its specific role in oocyte maturation and early embryonic development (Xu et al., 2016).

During mammalian oocyte and early embryonic development, a group of maternal effect genes were found to play critical roles in regulating oocyte maturation, fertilization, and early embryonic development by modifying multiple processes, including maternal mRNA clearance, chromatin remodeling, and zygote genome activation (Kim and Lee, 2014). Among them, a multiprotein complex called SCMC was recently identified in mice and was proven to be essential for mouse cleavage-stage embryogenesis (Lu et al., 2017). The knockout mouse models of SCMC members were infertile and displayed embryonic developmental attest at the two-cell stage (Tong et al., 2000; Li et al., 2008; Yurttas et al., 2008; Yu et al., 2014). Padi6 was considered to be a member of SCMC, and eggs ovulated by female mice lack of *Padi6* could be fertilized but could not develop beyond the cleavage stage (Wright et al., 2003; Esposito et al., 2007).

In our previous study, we found that the *PADI6* mutations could lead to the reduction of PADI6 expression in patients’ oocytes, which could impair the ZGA process and finally cause the developmental arrest of embryos (Xu et al., 2016). In this study, due to the limitation of clinical samples, we did not investigate the *in vivo* expression of *PADI6* variants in patients’ oocytes. Instead, we conducted the *in vitro* expression assay and compared the expression level of p. Cys163Arg and p. Trp475* variants with the wild-type and the pathogenic variants identified in our previous study: p. His211Gln, p. Gln324*, p. Gln381*, and p. Glu670Glyfs*48. The two variants displayed a significant reduction in protein expression, which was similar to the previously identified variants. Additionally, the mRNA levels were significantly reduced in p. Trp475*, p. Gln324*, and p. Gln381* variants, suggesting that the premature termination codons might cause the degradation of mRNAs by nonsense-mediated mRNA decay (NMD), thus resulting in significantly reduced truncated proteins eventually. Because the p. Cys163Arg variant was a missense variant, we performed a 3-D structural prediction to provide supporting evidence of the pathogenicity of the variant. The wild-type Cys163 was located in the central core area of protein, and replacement of the noncharged cysteine residue by positive-charged arginine could alter the charge balance of the local area, which might lead to misfolding and degradation of the protein. We speculated that the variants could cause a significant reduction of PADI6 in oocytes, which might destroy the SCMC structural and functional stability, further affect the ZGA process of the early embryos, and finally lead to developmental arrest. Therefore, an effective supplement of PADI6 protein or mRNA in oocytes might rescue the phenotype, which is highly promising to investigate knockout mice in further study.

To summarize, we identified two novel homozygous variants (c.487T > C [p.Cys163Arg], and c.1425G > A [p.Trp475*]) in *PADI6* in two females experiencing recurrent embryo developmental arrest. Our study expands the spectrum of genetic defects in female early embryonic arrest, and *PADI6* is worth being screened in females with similar phenotypes.

## Data Availability

The data sets presented in this study can be found in online repositories. The name of the repository and accession number can be found below: China National GeneBank DataBase (CNGBdb), https://db.cngb.org/, CNP0002647.

## Supplementary Material

Supplementary Figure S1 Expression pattern of four candidate genes identified from family 1. **(A)** Expression dynamics of four candidate genes according to our in-house RNA-Seq data. GV: germinal vesicle oocyte; MI: metaphase I oocyte; MII: metaphase II oocyte. **(B)** Expression dynamics of four candidate genes according to single-cell RNA-Seq data published by Yan et al (Yan et al., 2013).

Supplementary Figure S2 Quantitative RT-PCR analysis of PADI6 mRNA expression in HEK-293T cells transfected with wild-type or mutant plasmids. ^**^, *p* < 0.01; ^***^, *p* < 0.001.
