# Supplementary material for: Novel Homozygous PADI6 Variants in Infertile Females with Early Embryonic Arrest
Source: Front Cell Dev Biol. 2022 Apr 1;10:819667. doi: 10.3389/fcell.2022.819667 (PMC9010549; doi:10.3389/fcell.2022.819667)
Supplement: Supplementary file 1 [file Table1.DOCX]

Supplementary Table 1. List of primers used to detect expression of PADI6.

| **Primer name** | **Sequence** |
| --- | --- |
| PADI6-RT-F | ATGCCGTTTGTGTGTTGGG |
| PADI6-RT-R | TCTCAGAAATCACCGTGTTGG |
| GAPDH-RT-F | GGAGCGAGATCCCTCCAAAAT |
| GAPDH-RT-R | GGCTGTTGTCATACTTCTCATGG |
