# Supplementary figures and images for: Novel Homozygous PADI6 Variants in Infertile Females with Early Embryonic Arrest
Source: Front Cell Dev Biol. 2022 Apr 1;10:819667. doi: 10.3389/fcell.2022.819667 (PMC9010549; doi:10.3389/fcell.2022.819667)

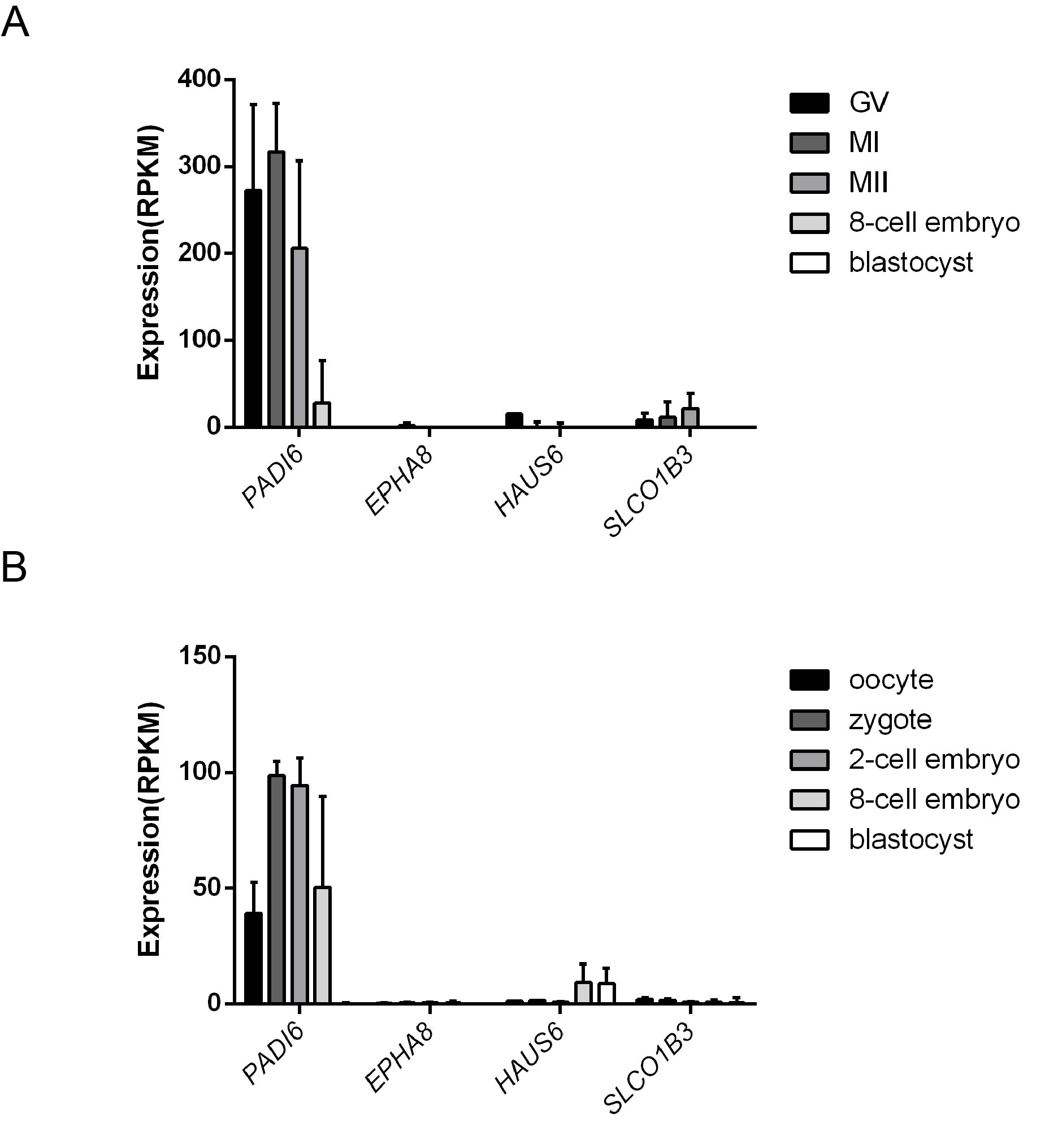

Supplement: Supplementary file 2 [file Image1.JPEG]

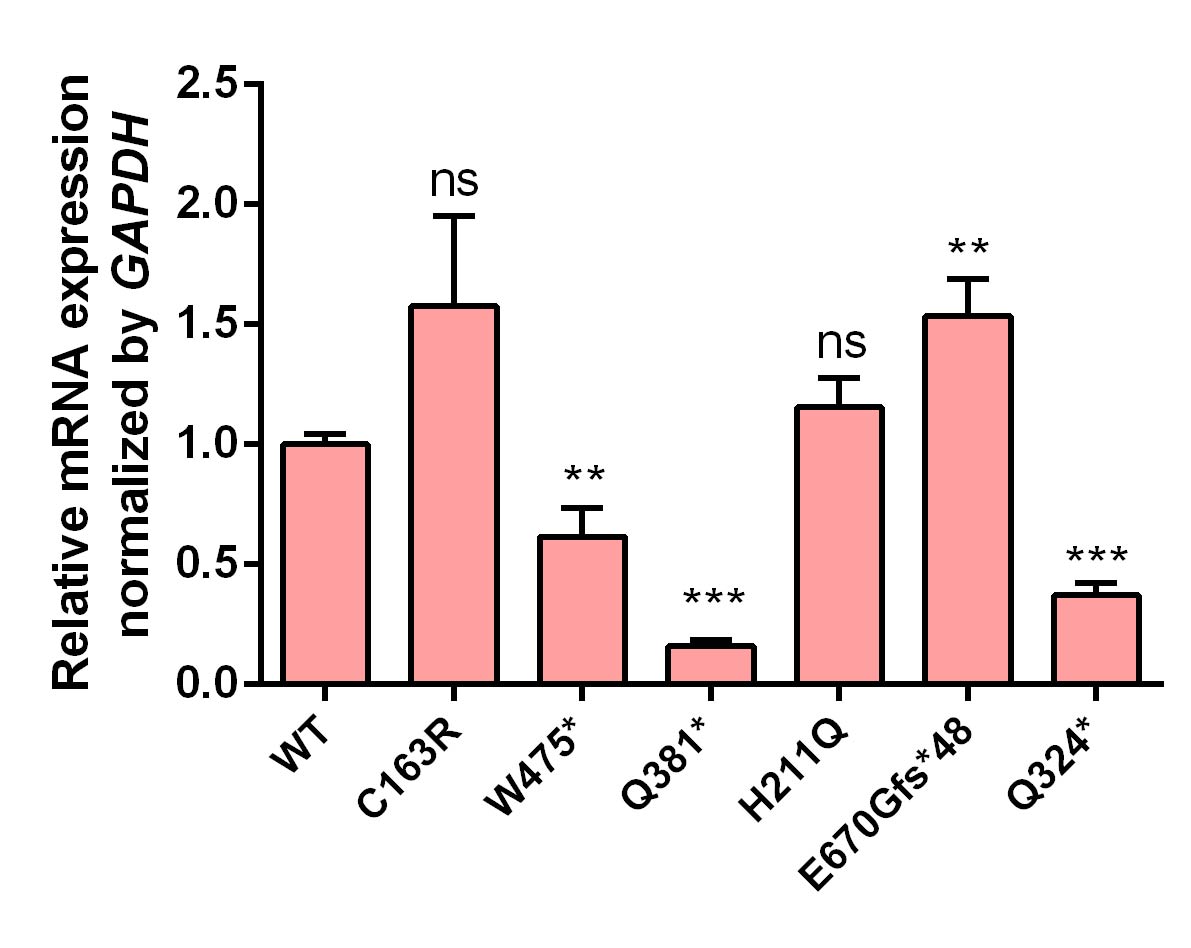

Supplement: Supplementary file 3 [file Image2.JPEG]
